# Marine-Derived Polymers in Ionic Liquids: Architectures Development and Biomedical Applications

**DOI:** 10.3390/md18070346

**Published:** 2020-06-30

**Authors:** Simone S. Silva, Joana M. Gomes, Luísa C. Rodrigues, Rui L. Reis

**Affiliations:** 1 3B´s Research Group, I3Bs- Research Institute on Biomaterials, Biodegradables and Biomimetics, University of Minho, Headquarters of the European Institute of Excellence on Tissue Engineering and Regenerative Medicine, Avepark, 4805-017 Barco, Guimarães, Portugal; joana.gomes@i3bs.uminho.pt (J.M.G.); luisa.rodrigues@i3bs.uminho.pt (L.C.R.); rgreis@i3bs.uminho.pt (R.L.R.); 2 ICVS/3B´s – PT Government Associate Laboratory, 4805-017 Braga/Guimarães, Portugal; 3 The Discoveries Centre for Regenerative and Precision Medicine, Headquarters at University of Minho, Avepark, 4805-017 Barco, Guimarães, Portugal

**Keywords:** marine polymers, ionic liquids, tissue engineering, membranes, hydrogels, sponges

## Abstract

Marine resources have considerable potential to develop high-value materials for applications in different fields, namely pharmaceutical, environmental, and biomedical. Despite that, the lack of solubility of marine-derived polymers in water and common organic solvents could restrict their applications. In the last years, ionic liquids (ILs) have emerged as platforms able to overcome those drawbacks, opening many routes to enlarge the use of marine-derived polymers as biomaterials, among other applications. From this perspective, ILs can be used as an efficient extraction media for polysaccharides from marine microalgae and wastes (e.g., crab shells, squid, and skeletons) or as solvents to process them in different shapes, such as films, hydrogels, nano/microparticles, and scaffolds. The resulting architectures can be applied in wound repair, bone regeneration, or gene and drug delivery systems. This review is focused on the recent research on the applications of ILs as processing platforms of biomaterials derived from marine polymers.

## 1. Introduction 

Natural polymers from marine resources have increasingly attracted attention in recent years, as they are abundant and biologically active when compared to polymers from other resources. In fact, marine sources such as crustaceans, seaweeds, and algae are enriched with polysaccharides such as agar, chitin/chitosan, alginate, and glycosaminoglycans, exhibiting interesting features and properties [1,2,3]. For instance, chitin acts as a structural material in the exoskeletons of crustaceans and insects. Such marine-derived biomaterials constitute a platform for the development of value chains with environmental and economic advantages. In fact, several marine polymers are entering the biomedical market due to their abundance and their intrinsic features, namely biocompatibility, biodegradability, and biological activity. Despite these advantages, some mentioned polysaccharides have limitations of solubility in water and most organic solvents, due to the strong intra- and intermolecular hydrogen bonds in their polymeric chains, which limit their processing and conversion into value-added matrices, e.g., membranes, fibers, nanomaterials, and scaffolds. Hence, searching for effective, ecofriendly, and feasible solvents is very important. 

Ionic liquids (ILs) are organic salts and an important green media [4], mainly explored in biopolymers processing, but also able to be extracted directly from their sources [5,6,7,8]. The interest in ILs occurs due to their excellent properties, such as very high thermal stability, recyclability, noninflammability, negligible vapor pressure, and miscibility in various solvents [4]. A vast range of available ILs can be tuned, combining different cations and anions, which would tailor their intrinsic features, such as viscosity, ionic conductivity, density, polarity, solvation power, hydrophilicity, and hydrophobicity [9,10,11]. Different researchers proposed distinct strategies that combine green chemistry principles with the use of biorenewable feedstocks, e.g., natural macromolecules envisioning the formation of 2D/3D matrices as innovative biomaterials [12,13,14,15,16,17]. Pioneering work on the use of 1-butyl-3-methylimidazolium chloride (Bmim)(Cl), an IL as a solvent for cellulose in relatively high concentrations (30–40% wt), was developed by Swatloski et al. in 2002 [18]. Their success in the dissolution of cellulose in ILs opened new avenues for the processing of other biopolymers [6,19,20].

Despite the advantages of ILs, their high cost is a major issue in large-scale applications, making their recycling an important issue to assure economic sustainability. Taking into account that ILs cannot be purified by distillation due to their low volatility, the recycling of ILs could be challenging. For their part, simple protocols based on the solubility of ILs in organic solvents have been developed [21]. 

**Figure 1:**
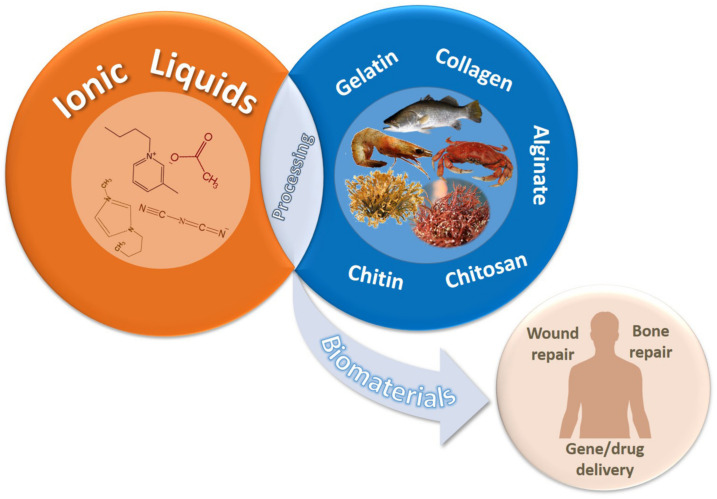
Strategies and biomedical applications of chitin/chitosan-based biomaterials prepared in ionic liquids (ILs).

Particularly, the use of ILs to extract marine polysaccharides like agarose, chitin, carrageenan from their sources, residues, or even waste is a sustainable approach that has been indicated as easy and highly efficient, as compared to the conventional methods of extraction [7,22]. Besides polymer extraction, many studies have also shown the potential of ILs as suitable platforms for the production of 2D and 3D-based marine polymers, namely gels, films, micro/nanoparticles, and sponges. Given the performance of these matrices, they have been proposed as wound dressing, drug delivery, bone repair, and gene delivery systems. Therefore, this review is focused on the overview of the properties, strategies, and biomedical applications of marine-derived polysaccharides processed in different ILs (see Figure 1). 

## 2. Dissolution of Marine-Derived Polymers Using Ionic Liquids 

The unique physicochemical properties and improved solvating power of ILs allow to dissolve a variety of polysaccharides through the disruption of hydrogen bonding. In particular, many ILs, such as 1-ethyl-3-methylimidazolium acetate ((Emim)OAc)) [23,24], 1-butyl-3-methylimidazolium acetate((Bmim)(OAc)) [25], N-butylpyridinium hexafluorophosphate (BPPF6) [26], 1-butyl-3-methylimidazolium chloride ((Bmim)(Cl)) [27], 1-ethyl-3-methylimidazolium ethylsulfate ((Emim)(C_2_OSO_3_)), 1-hydrogen-3-methylimidazolium hydrogen sulfate ((Hmim)(HSO_4_)) [28], 1-Butyl-3-methylimidazolium tetrafluoroborate ((Bmim)(BF_4_)) [29], 1-ethyl-3-methylimidazolium chloride ((Emim)(Cl)) [30], and 1-ethyl-3-methyl-imidazolium ethyl sulfate ((Emim)(EtSO_4_)) [31] have been reported in the literature as capable of dissolving many marine polysaccharides, such as alginate, chitin, chitosan, collagen, and gelatin, among others. 

The abundance of marine microalgae and the skeletons of crustaceans is large, but its poor solubility in conventional solvents restricts the efficient extraction of its value compounds. Nevertheless, there are some reports on ILs as extraction mediums for polysaccharides from marine residues [5,7,22,32,33]. Details about the structures, properties, and strategies involving the use of ILs on the dissolution and processing of selected marine polymers are made in the following sections. 

Moreover, the interaction of the matrices, especially of proteins with ILs, has been assessed with various ILs, being the dominance of anionic interactions considered mostly responsible for governing stabilization [34,35,36,37,38]. Considering collagen, ILs based on imidazolium, phosphonium, and ammonium had destabilizing effects because of the chaotropic of anions resulting in collagen structural degradation rather than the strengthening of interactions, although choline dihydrogen phosphate (Ch)(DHP) stabilized the collagen structure [37,39,40,41]. In another work, choline amino acid-based ILs also demonstrated a destabilizing effect at the molecular and fibrillar levels, due to competitive hydrogen bonding between its molecules [37]. Either (Ch)(DHP) and choline amino acid-based ILs belong to the biocompatible ILs (bio-ILs) family. Bio-ILs emerged due to the need to develop more biological and environmental-friendly compounds that will allow extending the use of ILs to a broader range of fields, preventing the associated toxicity issues. The synthesis of bio-ILs is mainly performed using the choline cation as the cationic counterpart; however, in recent years, more synthetic strategies have been developed [42]. Furthermore, the role played by the cation can not be neglected; according to Mehta et al., significant physicochemical impacts, including on thermal denaturation, were observed for different aqueous solutions of imidazolium chlorides ((Emim)(Cl), (Bmim)(Cl)), and 1-decyl-3-methylimidazolium chloride ((Dmim)(Cl])) [40]. 

### 2.1. Alginate 

**Figure 2:**
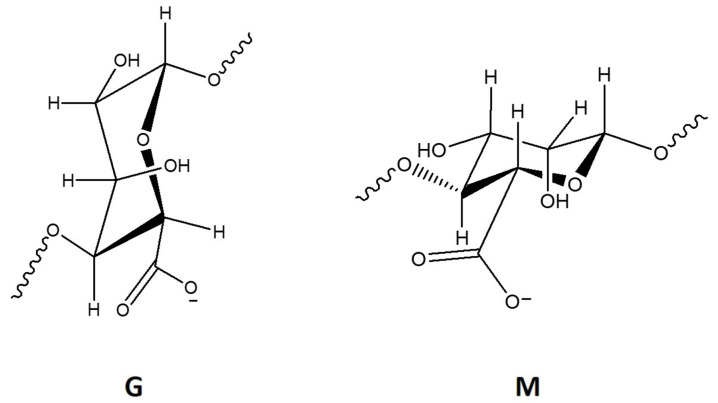
Chemical structure representation of alginate’s α(1-4)-linked l-guluronic acid (G) and β(1-4)-linked D-mannuronic acid (M) units.

Alginate is a linear polymer with a high abundance in nature [21]. It is present in the cell wall of brown algae, playing not only a structural function but also being involved in ionic exchange mechanisms. Alginate is an unbranched polysaccharide composed of β(1-4)-linked D-mannuronic acid (M) and α(1-4)-linked L-guluronic acid (G) (Figure 2), which are stereoisomers and differ in the composition of the carboxyl group [1,43]. M and G units can be present in blocks of (M and G) or mixed (MG) [1]. 

Alginate is water-soluble, being well-known for its gelling capacity [21]. The physicochemical properties of alginate, as well as its gelling ability, are strongly influenced by its structure, which is dependent on the variability in the ratio and sequence of the M and G units. Other factors influencing the alginate’s gelling capacity are the experimental conditions, including the solutions viscosity and the used gelation agent concentration, as well as the molecular weight. The most commonly used gelation agents are calcium and other divalent ions [1]. G monomers play a vital role in the ionic gelation mechanisms, forming ionic bonds induced by the presence of divalent ions [44]. G-rich alginate promotes the formation of more stiff and transparent gels, while M-rich alginate originates more flexible gels [45]. Alginate’s biocompatibility, low toxicity, and low cost have been boosting its wide use in areas, including food, cosmetics, pharmaceutical, and biomedical industries [1,21].

Despite alginate’s remarkable and exclusive features, depending on the target application, it generally lacks the desired physicochemical properties. Therefore, ILs are often used in combination with alginate to improve these properties. Some works have reported the development of electrolytes using alginate and ILs, with applications in electrochemistry and catalysis [21,26]. Ding and coworkers were able to develop a biosensor and biocatalyst of horseradish peroxidase using the IL N-butylpyridinium hexafluorophosphate (BPPF_6_) and sodium alginate [26]. These biosensing systems took advantage of the intrinsic ILs’ electrochemical properties, which allow a direct electron transfer. The produced film revealed to have good detection precision, bioactivity, storage stability, and reproducibility, suggesting the extension of its application to other enzymes. This is relevant, since the direct electrochemistry of redox proteins may help to understand the electron transfer mechanisms in the biological systems. Since ILs are great solvents for a wide range of other natural polymers, they also have been used for the preparation of composites consisting of alginate and other polymers, such as chitin [25]. In 2014, Shamshina et al. produced wound care dressings using chitin-calcium alginate composite fibers spun from an IL (Emim)(OAc) solution [25]. The produced fibers presented strength and water absorption, which met the technical specifications for wound care and allowed to accelerate the wound recovery, even though optimizations were needed.

### 2.2. Chitin 

**Figure 3:**
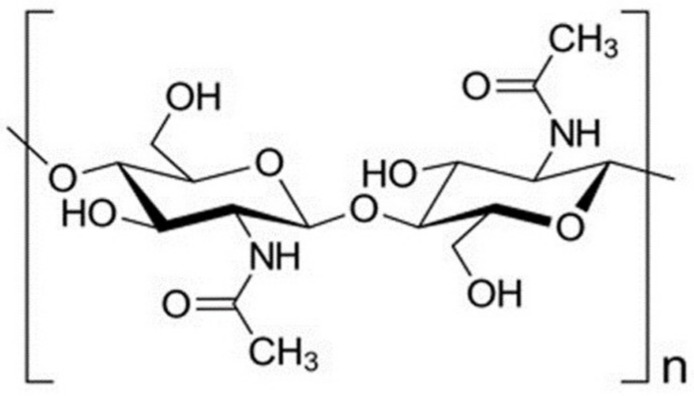
Chitin chemical structure representation.

Chitin is the second most abundant natural polymer, just after cellulose [46]. It is found in the shell of crustaceans, squid pens, fungi, and cuticles of insects [46,47]. Structurally, chitin is composed of a long chain made up of β (1→4)-linked primary units of N-acetyl D-glucosamine [48,49,50] (Figure 3). Chitin is characterized by its degree of acetylation (DA), which is defined as the ratio of 2-acetamido-2-deoxy-d-glucopyranose to 2-amino-2-deoxy-d-glucopyranose structural units, which is typically 0.90, indicating the presence of about 5-15% of amino groups due to deacetylation that might occur during chitin extraction [50]. 

Depending on the source, chitin can be characterized mainly by α- and β-forms [49,51]. In both types of chitins, the chains are organized in sheets and held together by intrasheet hydrogen bonds. However, β-chitin (e.g., from squid pens) presents weaker intermolecular bonds as compared to the α-chitin structure (e.g., from crabs and shrimp shells), which may explain its higher affinity for solvents and higher reactivity. Chitin is a highly crystalline polysaccharide due to the strong intra- and intermolecular interactions, namely hydrogen bonds derived from acetamido groups—more specifically, between C = O and NH groups of the adjacent chitin chains, established between the polymeric chains, which makes difficult its dissolution and, consequently, its processing. 

The biomedical potential of chitin is enormous, not only due to its abundance but, also, due to its biocompatibility, nontoxicity, and suitability for wound and burn healing. Despite the annual production of the biomass, the utilization of chitin as a raw material is limited due to its lack of isolation and solubility. In fact, chitin has shown difficulties using traditional solvents. A large volume of research has demonstrated the isolation and efficient dissolution of chitin, followed by the production of chitin-based matrices for many applications. Rogers et al. have shown that high molecular regenerated chitin can be extracted directly from shellfish waste (yield of 46%), and it could easily be processed into nanomats through electrospinning and ILs ((Emim)(OAc)) [5,23,24]. In another study, chitin was directly extracted from crab shells by using an ionic liquid, 1-allyl-3-methylimidazolium bromide ((Amim)(Br)) [52]. They indicated that ILs tend to extract chitin without addition to strong acid and/or base. Although there are promising findings on the use of ILs to dissolve chitin, little research has been performed on the influence of IL composition and polarity into the chitin dissolution mechanism. It seems that the requirement is of a higher polarity and more basic anions, e.g., acetate, probably due to the higher number of hydrogen bond donors and acceptors [23,53,54]. The studies suggested that acetate ions gave origin to weak conjugate acids able to interact with H-bonds of chitin, destroying them and leading to chitin crystal dissolution. Therefore, acetate ions can be more effective than chloride or dimethylphosphate anions. Studies involving a molecular dynamics (MD) approach to evaluate the dissolution of chitin crystals in imidazolium-based ILs revealed that the solubility of chitin can be correlated with the number of intermolecular hydrogen bonds by acetamido groups in the chitin crystal [55]. The data also proved that mixing a small amount of 2-bromoethyl acetate, as a bromide generator, with (Amim)(Br)can enhance chitin solubility. Besides the chosen solvent, parameters such as the degree of acetylation, pH, and chain molecular weight of chitin can affect its solubility, and it should be considered to understand the ability of this polymer to solubilize with ILs. 

Moreover, different chitin-based materials processed through ILs have been prepared, such as chitin ion gels made with (Amim)(Br) (9.1–10.7 wt%) [56] that were used to produce highly entangled nanofibers with added functional components and modulated material morphology, which may find potential applicability in membrane preparation [57], biomedical and tissue engineering applications [58,59], biosensors [60], or carbon capture sorbents [61]. 

### 2.3. Chitosan

**Figure 4:**
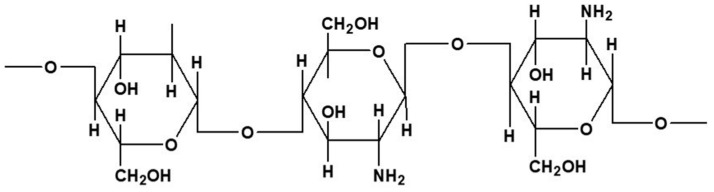
Chitosan chemical structure representation.

Chitosan is a deacetylated derivative of chitin, and it has also been extensively studied for several purposes in food science, agriculture, environmental, textile, and biomedical fields [48,49]. It is composed of β-(1-4)-linked d-glucosamine (deacetylated monomer) and *N*-acetyl-d-glucosamine (acetylated monomer) units (Figure 4), in which the glucosamine backbone holds a high number of available amino groups that can be protonated. It is commercially available in a broad range of molecular weights and degrees of deacetylation. Moreover, amino groups of chitosan have a pKa value close to 6.5, which confers it with solubility in weak acid solutions, namely dilute acidic solutions of acetic, citric, and lactic acids [48]. Therefore, its charge density is dependent on the pH and the degree of deacetylation. Both the degree of deacetylation and molecular weight determine the properties of chitosan, e.g., biodegradability, biocompatibility, and solubility. 

Similar to chitin, chitosan is extremely difficult to dissolve in water and most of the conventional organic solvents due to its strong intra- and intermolecular hydrogen bonds. Therefore, the search for sustainable solvents of chitosan has drawn wide attention to overcome the environmental issues related to acid-base treatments. Specifically, the role of ILs for the dissolution of chitosan has been evidenced with different degrees of success. So far, the existent similarity between cellulose and chitosan and the acquired know-how achieved for cellulose processing in IL have acted as a starting point for many strategies for chitosan processing in ILs. The research performed involved the elucidation of the effect of the IL composition, dissolution conditions (temperature and polymer concentrations), and water content in the chitosan hydrogen bonds’ disruption promoted by ILs. Based on many studies, some ILs, including those with chloride, formate, and acetate as anions and 1-allyl-3-methylimidazolium ((Amim)), 1,3-dimethylimidazolium ((Dmim)), 1-hydrogen-3-methylimidazolium ((mim)), and 1-butyl-3-methylimidazolium ((Bmim)) as cations, as well as their mixtures, have been investigated as solvent and reaction mediums for chitosan [17,19,62,63,64]. The performance of a series of imidazolium-based ILs on chitosan dissolution demonstrated that (Bmim)(OAc) IL is the most efficient one [62]. Following the findings, the ability to dissolve chitosan follows the order: (Bmim)(OAc) > (Emim)(OAc), (Bmim)(OAc) > 1-hydrogen-3-methylimidazolium hydrogen acetate ((Hmim)(OAc)) > 1-octyl-3-methylimidazolium acetate (Omim)(OAc), and (Bmim)(Ac) > 1-butyl-2,3-dimethylimidazolium acetate (Bmmim)(OAc). The solubility of chitosan decreased with the increase of the water content at temperatures below 110 °C—after which, the values were resembled, probably due to water evaporation, while other studies observed an enhanced chitosan solubility with the increase of the dissolution temperature, e.g., from 50 °C up to 150 °C [64,65]. This effect is mainly associated with a change of the transport properties of the ILs and, simultaneously, to the evaporation of residual water from the system that is considered as antisolvent. Considering all the mentioned variables, the imidazolium-based ILs are advantageous for chitosan dissolution [65]. 

(Bmim)(Cl) has been used as an environmentally friendly solvent to prepare chitosan/cellulose biocomposites sorbents for industrial effluent treatments [22]. Moreover, due to its strong interactions with negatively charged entities, including lipids and proteins, IL/chitosan-based aerogels are used to stabilize the complexes, with DNA fragments being good choices for gene delivery systems [66].

### 2.4. Collagen 

Collagen is the major supportive component of connective tissue, making up about 25–35% of the whole-body protein, being present in bone, tendon, teeth, skin, ligaments, and cartilage [67,68,69,70,71]. Collagen-based products, with high added values and low environmental impacts, have gained interest from the research community, as they can be obtained through the conversion of low-cost by-products. The preferential sources for collagen extractions are terrestrial mammals like cows, pigs, and sheep, due to the high-sequence homology with human collagen [72]. However, different concerns are associated with mammalian collagen, such as the trigger of an immune reaction (around 3% of the population), the transfer of zoonosis, and cultural or religious concerns associated with the use of porcine and bovine collagen, which further restrict its application [73]. The use of marine-derived collagen significantly restrings those concerns, being free from religious concerns and intrinsically showing a lower threat of transmissible diseases. Therefore, the possibility to valorize the fish byproducts (e.g. fish skin and scales) derived from the largely available polluting by-products from the fish processing industry as collagen sources makes marine-derived collagen ecofriendly and particularly attractive in terms of profitability and cost-effectiveness [72,74,75].

Considering the collagen molecule chemical composition, it can be described as a protein containing three polypeptide chains, each of which is composed of one or more regions containing an uninterrupted repeat of Gly-X-Y sequences, where X and Y can be any other amino acid residue. The sterical constraints due to proline and hydroxyproline cause the collagen regions with this tripeptide repeat to adopt three left-handed polypeptide chains (called α helices), which self-assemble to form at least one right-handed triple-helical domain [72,76,77], providing not only structural support for cells but, also, acting as an important regulator of cell behavior [78]. Collagen can be isolated from natural products, being relatively nonimmunogenic and, consequently biocompatible, opening the possibility to use it in a wide range of applications in commercial fields, including food [79,80], cosmetics [80,81,82], and medicine [72,83,84,85,86]. However, collagen application is tremendously limited by the strong inter- and intramolecular hydrogen and ionic bonds, van der Waals’ forces, and hydrophobic bonds between the polar and nonpolar groups, which have extremely difficult collagen dissolution and consequent processing [71]. The hydrogen bonds formed by proline and hydroxyproline have a fundamental role in stabilizing the triple helical structure in physiological conditions, preventing chain free rotation [87]. Those bonds can be broken upon denaturation through thermal or chemical treatments, significantly impacting the collagen properties as it transforms collagen into a random coil form known as gelatin [72]. Several efforts have been made to disperse or dissolve collagen, preserving its native structure and simultaneously improving the content of collagen in the solution, as it is insoluble in organic solvents and only a low percentage is soluble in dilute acids and alkalis [88]. Different strategies have been employed to improve collagen dissolution. Poluboyarov et al. [89] reported values reaching 19.5g/L after five days for the combined effects of mechanical (ultrasonic and laboratory mixer) and enzymatic treatments, and Qi et al. [78] reported the achievement of a 10% (1 g/10 mL) collagen dissolution in a NaAc/HAc buffer solution. Another successful approach is the dissolution of collagen using ionic liquids (ILs) as a solvent. In this approach, the IL interacts with collagen by a hydrogen bond, promoting its dissolution [38,71,86]. Imidazolium-based ILs have brought about significant changes at the higher structural hierarchical level of collagen, developing a different hierarchical ordering [40]. Phosphonium and ammonium-based ionic liquids have a destabilizing effect on collagen [35,36]. On the other hand, (Ch)(DHP) IL stabilized collagen by exerting an electrostatic force on collagen, and due to its biocompatibility, has potential as biocompatible crosslinkers [90]. Collagen-based biomaterials prepared using choline salt, as crosslinkers, exhibited good cell viability and adhesion properties, as required for biomedical implantable applications [91].

### 2.5. Gelatin 

**Figure 5:**
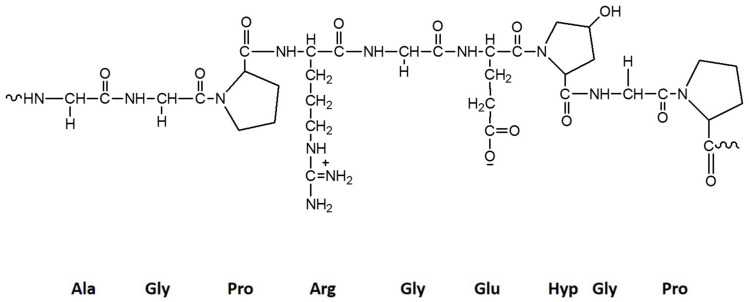
Basic chemical structure of gelatin (Ala—alanine, Arg—arginine, and Glu—glutamate).

Gelatin is the partially hydrolyzed form of collagen. Although their sources are bovine and porcine skin, some studies demonstrated their extraction from marine sources such as sponges and fish skin [43]. The chemical composition of gelatin depends on the source, but hydrophobic amino acids like proline (Pro), hydroxyproline (Hyp), and glycine (Gly) are more likely to be present in gelatin. The general primary sequence is given by (Gly-X-Pro) and (Gly-X-Hyp), in which X represents other amino acids [92]. In Figure 5 is presented the model structure of gelatin. 

Gelatin has a high solubility in water, as well as in many ILs [31]. The dissolution of gelatin in water occurs after the polypeptide strands in its structure undergo a coil-helix transition, and it happens at 30–35 °C [93]. Gelatin’s amino acid content, which includes positively charged (lysine and Arg, 7.5%); negatively charged (Glu and aspartic acid, 12%); neutral (Gly, Pro, and Hyp, 58%); and hydrophobic (leucine, isoleucine, methionine, and valine, 6%) amino acid residues, promotes a different set of interactions with solvents or electrolytes [31]. This polymer is inexpensive, biocompatible, and biodegradable and interacts strongly with molecules that are soluble in aqueous media [93]. 

The gelatin and ILs combination strongly contributes to a broader use of this polymer. ILs can provide different physical-chemical properties and, also, change the gelatin microenvironment, being able to address one of gelatin’s limitations, which concerns the entrapment of poorly water-soluble molecules [93]. The combination of ILs with gelatin is often used for the production of ion gels (IGs) [31,93,94]. This is mainly possible due to the expected set of electrostatic, hydrophobic, and H-boding interactions between the biopolymer and ILs, which lead to the formation of IGs [31]. This gelation process is based on a good compromise between the retention of the IL and its fluidity inside the polymeric network [93]. This technology allows the production of versatile and conductive gels that can be molded into different shapes using different methodologies. The produced IGs are usually simpler than the common solid polymer electrolytes and exhibit improved conductivities, which boosts its use as substitutes for the existing solid-state polyelectrolytes in energy devices [94]. Moreover, these electrolytes may often be used as printable “inks” [31,94]. Several authors have been using these IGs for the development of biosensing devices, namely for the immobilization of oxidoreductases, such as glucose oxidase (GOD) and horseradish peroxidase (HRP) [93,95,96]. Lourenço et al. prepared glucose paper test strips by the physical deposition of gelatin-1-ethyl-3-methyl-imidazolium ethyl sulfate ((Emim)EtSO4) containing GOD and HRP, as well as color-generating precursors [95]. The entrapment of GOD and HRP in the IGs show lower activity than for the free enzyme—in both cases, however, with excellent storage stability at 4 °C for a period of two weeks. Moreover, the immobilization of color-generating precursors in combination with the enzymes in the composite materials demonstrates that it can be used for the development of cheap and straightforward glucose paper test strips, with a quick response in less than one minute. Furthermore, these systems are used as drug delivery systems either by the functionalization of IL-based polymer gels by the incorporation of the active principle or by exploring the IL as the active principle ingredient [93,97]. Moreover, the use of ILs as the substituent of the chemical crosslinkers may allow to form relatively nonsoluble networks and significantly expand gelatin applications, since some of these polymers’ limitations are extensive swelling, rapid dissolution, and drug release [93]. In 2019, Maneewattanapinyo and coworkers were able to develop a lidocaine–diclofenac-IL drug–loaded transdermal patch using the polymers gelatin/poly(vinyl alcohol), where the IL worked as the active pharmaceutical ingredient [97]. The developed biomaterial presented good physicochemical properties and showed to be viable to be used in pharmaceutics, mainly due to the control release of both lidocaine and diclofenac. Moreover, the developed patch presented good stability over the study period of three months when kept at 4 °C or under ambient temperature.

### 2.6. Other Marine-Derived Polymers

Besides the mentioned polymers above, the use of ILs to solubilize or even extract the medium for other marine-derived polymers such as carrageenan [33,98], agarose [6,7,30], and chondroitin sulfate [28] have also been investigated. Some authors explored the ability of ILs as a medium for the efficient extraction of agarose, the main agar constituent, from red algae (Rhodophyta). For that purpose, different ILs (1-ethyl-3-methylimidazolium acetate, (Emim)(OAc), choline acetate, (Ch)(OAc), and 1-ethyl-3-methyl imidazolium diethyl phosphate, (Emim)(Dep)) and heating or microwave irradiation were applied in the process [7]. As compared to conventional methods, a very high extraction yield of good quality agarose (as high as 39 wt%) was obtained. In other studies, the versatility of ILs combined with the morphological adaptability of the agarose was investigated to the formation of agarose-based highly soft ion gels [6]. In other approaches, the extraction of ĸ-carrageenan from the red marine macroalgae Kappaphycus alvarezii was studied, applying an ionic liquid-assisted subcritical water (SWE) [33]. The findings showed that the SWE with a (Bmim)(OAc) IL catalyst exhibited the highest percentage yield, probably due to its high depolymerization and dissolution ability. Additionally, the formation of carrageenan (k-, ι-, and λ) combined with cellulose was achieved using (Bmim)(Cl), where λ-carrageenan gave a better miscible composite gel with the IL [98]. In a similar approach, chondroitin sulphate, a macromolecule classified as glycosaminoglycan, was blended with chitosan, using (Hmim)(HSO_4_) as an appropriate solvent to create blended hydrogels. Those hydrogels showed excellent stabilities in a wide pH range (1.2–10) and excellent biocompatibility with epithelial cells. 

## 3. Development of Marine-Polymeric Architectures via Ionic Liquids

Many 2D and 3D-based architectures have been produced using the dissolution of marine polymers with different ILs at moderated high temperatures, followed by cooling the polymer/IL solution to low temperatures (4–25 °C), promoting the formation of weak gel-like materials (ion gels), films, and hydrogels (see examples in Table 1 and Table 2). By soaking those gels in water or ethanol and/or applying a suitable processing technique, e.g., freeze-drying, solvent casting, or electrospinning on the polymer/IL-based solutions, sponges, films, hydrogels, or nano/microspheres can be produced. Considering that some toxicity studies on ILs suggested that they exhibit a certain level of toxicity, their total or partial removal from the structures should be made. More details about the production of different matrices involving marine polymers and ILs are described in the following sections. 

### 3.1. Films and Hydrogels 

**Figure 6:**
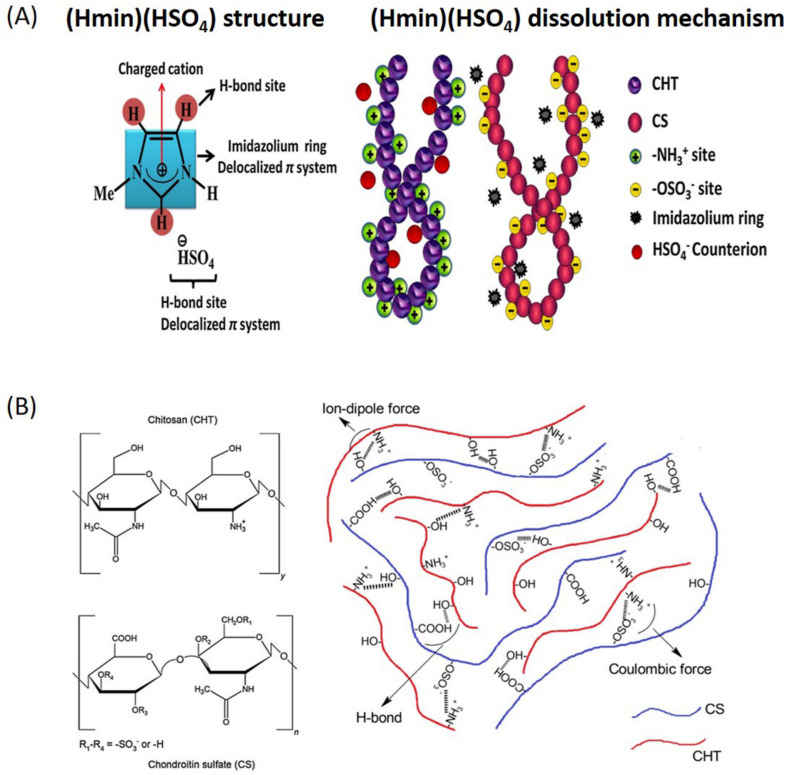
(**A**) (Hmim)(HSO_4_) structure and its solvation capacity over the biopolymers and (**B**) the polysaccharide structures (left panel) and chitosan/chondroitin sulfate (CHT/CS) arrangement (right panel). Reprinted from [28], Copyright 2017, with permission from Elsevier.

The ability of ILs in dissolving marine-derived polymers have been used to create films and hydrogels. The general procedure involves the dissolution of the polymers at a high temperature and gelation at room temperature with or without the use of specific molds, followed by immersion of the polymer/IL gels in solvents such as ethanol, acetone, or isopropanol. The choice of those solvents is related to their miscibility with the ILs, which, in turn, promotes the IL removal from the structures. Chitin films with tunable strength and morphology were designed by different drying methods, e.g., a simple casting method from a solution from (Emim)(OAc) or sc-CO_2_-drying [99]. The chitin films were able to load and release caffeine, which was used as a model drug, indicating that they may have potential as drug-releasing membranes. It was shown that combinations of marine polymers with other polysaccharides, proteins, or even inorganic particles, using a common IL as the solvent, can be used to mimic the naturally occurring environment of certain tissues. Chitosan/silk fibroin (CSF) hydrogels were prepared in (Bmim)(OAc) as a common solvent and a soxhlet extraction with ethanol for IL removal [14]. The CSF exhibited viscoelastic behavior, lamellar structure, and rubbery consistency and, also, supported the adhesion and growth of primary human dermal fibroblasts. In another study, chitosan/chondroitin sulfate hydrogels were prepared in (Hmim)(HSO_4_). Figure 6A depicts the IL structure, as well as the polymer dissolution mechanism proposed. The (Hmim)(HSO_4_) solvent displayed a pH of 2.5 at 25 °C, and thus, the NH_2_ ionization of chitosan can occur according to the following reaction: $R-NH_{2}+H^{+}\to \;R-NH_{3}^{+}$, whereas the pKa amino site is roughly 6.5. The coulombic, H-bond, ion-dipole, and London forces between CHT/IL foster CHT-dissolving (Figure 6A, right panel). Regarding the CS solution, there is an equilibrium of charges [-OSO_3_H] ≈ [-OSO_3_^−^] under IL (pH 2.5) due to the pKa for −OSO3H being approximately 2.6. The CS dissolving was similar to that designated for CHT (6A, right panel). Thus, polyelectrolyte complexes (PECs) are inherently established by coulombic, H-bonds, and ion-dipole forces (Figure 6B, right panel). The chitosan/chondroitin sulfate hydrogels achieved excellent stability in the 1.2-10 pH range, considerable swelling abilities, and were devoid of toxicity towards the normal healthy kidney epithelial and epithelial colorectal adenocarcinoma cells [28]. Moreover, chitosan films with the potential to be used as drug delivery systems were developed using the bio-IL (Ch)(DHP) and the cholinium salt choline chloride [100]. The use of the bio-ILs provided the films with enhanced drug-release profiles, which associated with their conductivities/impedances, as well as pH sensitivities, allowing the development of biodegradable and biocompatible responsive drug delivery systems. In another study, choline nitrate (Ch)(NO_3_) was used in combination with chitosan to produce a thin-film polymer gel electrolyte [101]. Besides their biocompatible and biodegradable features, the developed films presented robust mechanical properties and high ionic conductivity, leading the authors to suggest their application as implantable medical devices, including cardiac pacemakers or biomonitoring systems. 

### 3.2. Nanomicrofibers and Nanomicroparticles 

Over the past decades, we have witnessed significant progress in marine-derived nanostructured materials. Nanofibrous materials have a remarkable potential, being useful in different applications such as drug delivery systems, tissue engineering scaffolds, wound dressing materials, antimicrobial agents, and biosensors. Due to their appealing physical and biological features, chitin and chitosan nanofibers have attracted the scientific community’s attention [19]. Pure high molecular weight chitin nanofibers were electrospun through a one-pot process in an [Emim][OAc] solution of chitin extracted from dried shrimp shells [23,24,102]. This strategy allowed achieving smooth, continuous chitin nanofibers directly from an extract that provided the optimal viscosity, concentration, and necessary entanglement density for electrospinning. Chitin-calcium alginate composite fibers were also prepared from a solution of high molecular weight chitin and alginic acid in (Emim)(OAc) by dry-jet wet-spinning into an aqueous bath saturated with CaCO_3_ [25]. Composite fibers presented consistent reproducibility and blend-homogeneity, meting the technical specifications (strength and water sorption) needed for wound care fiber applications; however, with the potential to be enhanced, envisioning different applications. High-tenacity chitosan fibers with excellent strength and initial modulus were generated with a dry-wet spinning technology from dissolved chitosan in binary IL mixtures of acidic and neutral IL of glycine hydrochloride (Gly·H)Cl and (Bmim)(Cl) [103]. The same procedures were used to produce chitosan-cellulose composite fibers with 9.4 wt% chitosan, which presented good mechanical strength and excellent thermal stability [104]. The same polymeric mixture was electrospun from an IL solution ((Emim)(Ac)) [105] to produce fiber films with the potential to be applied as antibacterial and antimicrobial agents to treat skin ulcers.

Collagen solutions were prepared in PBS containing different ration of ILs—respectively, 1-ethyl-3-methylimidazolium bromide ((Emim)(Br), 1-ethyl-3-methylimidazolium chloride ((Emim)(Cl)), or (Emim)(OAc) [106]. The thermal stability of the designed collagen fibril was significantly enhanced when the self-assembling was carried out in the presence of ILs, promoting as well the improvement of the viscoelastic properties of the collagen gel.

Microdroplets in ILs as unique interfaces led to the development of simple and rational methods for preparing biopolymer-encapsulated protein microcapsules [63]. The conventionally used methods for protein-based particle preparations have been employed (emulsification, desolvation, coacervation, and electrospray drying); however, other alternative strategies (template method, microfluidic technology, etc.) have been used to overcome the limitations associated with conventional approaches as low yields, low control of particles features (size distribution and shape), and collagen denaturation. Protein-based micro- and nanoparticles present high biodegradability and low thermal and mechanical stability, which lead to collagen chemical modification (maintaining its native structure) or the combination with other biopolymers or synthetic polymers or even inorganic materials, also allowing to increase the system functionality [107,108] and to modulate their properties according to the desired application. Thus, several collagen-based micro- and nanoparticles were developed with different biomedical applications (tissue engineering, imagistic/diagnosis, and drug/gene delivery) [109].

Oil-in-water microemulsions were used to prepare protein microcapsules (3-40 µm) [63,110]; however, the inner oil droplets are not suitable to dissolve water-soluble guest biopolymers. This issue can be overcome through the use of ILs, with the advantage that the microcapsules formed in the IL phase can be easily extracted to the aqueous phase after consecutive crosslinking and surface modification reactions [63,111,112,113]. Modifications were introduced into the emulsification method to improve the delivery kinetics, maintaining the collagen meshwork biocompatibility as the replacement of chemicals for photochemical crosslinking or the use of self-assembling collagen fiber reconstitution [114]. However, the emulsion method remains to present poor control of the particle shape and size, as well as a reduced loading level [109], which leads to the exploitation of other strategies to produce micro- or nanoparticles. 

Metal nanoparticles attract significant attention based on their properties, as they are reported to be monodispersed and non-agglomerated as a result of ionic liquid stabilization [112]. Polymers added to the nanoparticle–ionic liquid dispersion promotes a partial coverage of the nanoparticle surfaces where polymer coils extend between the particle surface, acting as bridges between nanoparticles through molecular contact. In higher amounts, the polymers can fully cover the nanoparticle surface to form an adsorbed polymer layer, which is responsible for steric repulsions between neighboring nanoparticles, “pushing” nanoparticles away from each other [112]. The generation of Ag_2_O nanoparticles in DSIL-gelatin sols showed uniform decorations of 50–100-nm size Ag_2_O nanoparticles over gelatin, wherein the imidazolium cation acted as a reducing agent [34]. This system presented good bactericidal activity on Gram-negative bacteria showing the potential to be used for food packaging, wound dressings, and other biomedical applications [34]. Hollow spheres were also fabricated according to template methodology, allowing to achieve gelatin particles with defined sizes and improved drug/protein loading and encapsulation efficiency, resulting in reservoir systems for the sustained delivery of proteins useful for different therapies [115]. 

Various self-organized structures based on nanoparticles are generated as a result of a balance of the intermolecular interactions between ionic liquids constituents and marine-derived polymers and proteins. Chitosan nanoparticles by ionic crosslinking with IL, which consist in self-assembling methods of adding (Bmim)(C_8_OSO_3_) or (Omim)(Cl) above the critical micelle concentration to an aqueous solution of chitosan, aggregate in a gelated complex [116]. The nanoparticles with diameters ranging from 300–560 nm and Zeta potential above +58.5 mV were formed due to the electrostatic and hydrophobic interactions established between chitosan and IL, being the IL aggregates used as templates for the structure build-up.

### 3.3. Scaffolds, Sponges, and Beads 

The combination of marine origin materials, in conjunction with green-processing technologies and solvents, has been proven to be effective for the development of scaffolds, sponges, and beads, with broad applications. Silva et al. [117] have successfully produced porous chitin aerogels by dissolving the polymer using the IL (Bmim)(OAc) by employing high-temperature stirring. After the mixture was gelified at room temperature and removed, and the IL was removed by supercritical fluid drying using a soxhlet extraction and SCF extraction using carbon dioxide/ethanol ratios. This procedure promoted the production of chitin aerogels with a porous and interconnected structure, large surface area, and low density. Moreover, chitin microparticles prepared in ILs were produced using a similar method to produce 3D constructs with the flexibility to adapt according to defect sites, osteoinductive behavior, and the potential use as controlled drug-release devices [58]. The sol-gel methodology was used to promote the formation of a silica network as a coating in the chitin beads, as well as a means to promote the IL removal from the beads, followed by the supercritical agglomeration method. 

The production of multifunctional composites using ILs, achieved by blends of different polymers or even inorganic particles (hydroxyapatite, HA), has also been a focus of study. In a work from 2013, Silva et al. [118] produced chitin–hydroxyapatite composites using (Bmim)(OAc) to dissolve chitin, followed by the addition of salt particles (salt leaching methodology) to promote pore formation and/or HA to induce osteoinductive behavior and drying by supercritical fluid drying. In a recent work from the same group, both chitin and *Antheraea pernyi* silk fibroin were dissolved using the same IL and used for the production of sponges from blends [13]. The produced sponges revealed to have good porosity, interconnectivity, and pore sizes values, presenting considerable swelling and adequate viscoelastic properties, making them promising candidates in cartilage regeneration. Composites with potential applications in bone tissue engineering were also formed by the dissolution of chitosan and cellulose in ILs with the addition of HA [27]. The produced structures presented good antimicrobial activity, the ability to deliver growth factors/drugs (from chitosan), and mechanical strength (from cellulose). 

## 4. Environmental and Biological Impact of ILs Used in the Development of Marine Polymer-Based Architectures

### 4.1. IL’s Recycling and Reuse

The recycling and reuse of the ILs increase the sustainability of the implemented processes while reducing the economical burdens sometimes related to ILs’ use, increasing the opportunity for the large-scale application of the developed methodologies [42]. The techniques used to recover and reuse ILs strongly depend on its application, considering that, usually, it involves a recovering or regeneration step, followed by a purification stage, to avoid the deterioration of the ILs [119,120]. The recycling of ILs used for the processing of marine-based biopolymers are regularly made by using an antisolvent (such as water and ethanol) that works as a coagulant to the dissolved polymer by solvating the ILs, constituting the recovering step. The purification step is usually performed by the distillation of the antisolvents from the ILs [120,121]. The use of water to recover the ILs after the polymer’s pretreatment is the most straightforward purification strategy [5,102,120]. Iqbal et al. prepared collagen-alginate-hydroxyapatite beds to be used as bone fillers, using (Emim)(Cl) as a solvent [122]. The IL was recovered from the water and CaCl_2_ mixture, used for the beads preparation, by rotary evaporation for water removal, followed by mixing with cold acetone to dissolve IL and precipitate out the CaCl_2_. After filtration, (Emim)(Cl) was obtained by the rotary evaporation of acetone, a process repeated many times, aiming to obtain a high yield of IL (95 ± 1). As well,(Bmim)(Cl), used as a green solvent to dissolve and synthesize the [CEL/CHT] composites, was removed from the composites by washing them with water. The IL was recovered by distilling the washing solution, from which the IL remained due to its high melting (>400 °C), with a recovery rate of at least 88% of the IL, being the proposed method considered as recyclable [123]. In another work, (Emim)(OAc) was used to directly recover high molecular weight chitin from raw crustacean shells [5]. Fibers were spun directly from the extract solution, and the IL recycling was carried out by evaporation of the aqueous wash. 

The use of aqueous biphasic systems in this process may contribute to a reduction of the energy needed for water evaporation since the kosmotropic salts pull some of the water present in the mixture. However, a further dewatering step using evaporation techniques is mandatory [5]. The dewatering strategies used for the purification of the ILs usually involve the use of deep vacuum (0.02mbar to 10mbar). Moreover, the use of heat or microwave-assisted eating proved to be helpful in that process, with the latter being 52 times more efficient than conventional heating [5]. When small molecules are formed, the product is usually recovered with a suitable volatile organic solvent (VOC) such as ethyl acetate, diethyl ether, or dichloromethane. This process is followed by the IL, and the additive or catalyst is used for recovery, and the water is removed through evaporation under high vacuum, and the products are used in the following catalytic steps [124]. Barber et al. extracted chitin from dried shrimp shells using (Emim)(OAc), followed by the chemisorption of CO_2_ in (Emim)(OAc) through the chemical reaction [121]. The use of CO_2_ proved to be an economical and energy-efficient method for the potential recycling of the IL by reducing the use of antisolvents and eliminating the need for using higher boiling coagulation solvents. 

Nevertheless, the choice and application of the recycling and purification method is always dependent on the starting material and contaminant level, as well as the chemical nature of the components. Moreover, and despite our knowledge not being reported in the literature for chitin or other marine polymers conversions, there are other methodologies that are independent of the mixture components [120]. One of the methods, which is patented by a Chemical Company, BASF, comprises the recovery of the IL through the formation of the distillable carbene, involving the imidazolium IL treatment with a strong base, which deprotonates the imidazolium cation at the C-2 position, forming 1,3-dimethyl-imidazol-2-ylidene carbene [125]. The formed carbene could be distillable out, and its reaction with the acid of the desired anion reforms the imidazolium IL. The described process can be applied after the desired products have been extracted from the IL, as well as after the dewatering of the IL/residues mixture. The use of membrane separation can also be considered as an effective strategy, including the commercially available pervaporation systems (PV). However, the efficiency of these systems is strongly dependent on the size and molecular weight of the mixture constituents, and it involves quite extensive work [102,120]. Every IL has different properties, which include different decomposition temperatures, hydrophilicity, and an optimal number of reuses [102]. Nonetheless, the persistent challenge is to find the best balance between the energy involved in the processes and its economical burdens. 

### 4.2. Biocompatibility

Although the IL platform suggests different pathways for the dissolution and processing of marine biomaterials into many matrices, as mentioned above, studies involving their in vitro/in vivo biocompatibility have not yet been fully explored. In fact, the application of 2D/3D-based marine biomaterials produced using ILs in the biomedical field is facing many challenges, since the implanted material could be influenced by the composition, architecture, and biocompatibility of the material. Considering that, ILs have been used in different approaches, such as a common solvent for combinations of marine polymers [25,123,126], proteins [14], or hydroxyapatites [122] as crosslinker agents [91] and modifiers [127] to render biomaterials with improved biocompatibility. An earlier report showed that the application of synthesized choline salts as crosslinkers on collagen-based biomaterials resulted in crosslinked materials with better cell growths compared to the sample crosslinked with glutaraldehyde [91], where the cells were found to be healthy and able to proliferate. In another approach [14], the use of ILs—particularly, (Emim)(OAc)—was useful as a common solvent in the combination of polysaccharides (chitosan) and proteins (silk fibroin) into hydrogels. Those hydrogels supported the adhesion and growth of primary human dermal fibroblasts, suggesting that they could be useful in skin regeneration approaches. In a similar study [86], an IL—namely, 1-methylimidazolium acetate ([Mim)(OAc))—facilitated the formation of alginate/collagen hydrogels with high hemocompatibility and satisfactory biocompatibility assessed by rat mesenchymal stem cells (rMSC), which rendered them as promising for skin dressings. Beyond that, another report showed that an IL, (Emim)(OAc), promoted the production of an electrically conducting chitin scaffold permissive for mesenchymal stem cell functions [127]. 

**Table 1:** Marine-derived polymers prepared using different ionic liquids.

Polymer/Matrix	Ionic Liquid/other Reagents	Process	Improved Properties	Potential Applications	References
Chitin	(Emim)(OAc)	Extraction/dissolution/ electrospinning	✓ smooth, continuous chitin nanofibers	Not defined	[23,24,102]
Chitosan	(Gly·H)Cl and (Bmim)Cl	Dissolution/dry-wet spinning	✓ nanofibers with excellent strength and initial modulus	Not defined	[103]
	(Bmim)(EtSO_4_) or (Omim)(Cl)	Ionic crosslinking	✓ NPs (diameters 300–560 nm) have controlled shape and size	Not defined	[116]
Collagen	(Emim)(Br)/ (Emim)(Cl)/ (Emim)(OAc)	Self-assembling	✓ fibril enhanced thermal stability; ✓ improvement of the viscoelastic properties of the collagen gel	Not defined	[106]
Collagen-based hydrogels	(Emim)(OAc)	Sol-gel transition	✓ [Emim][OAc] promoted high mechanical strength, degradation, resistance, and anti-inflammation effects.	Tissue engineering and cancer therapy.	[128]
Gelatin Microcapsules	(Bmim)(BF_4_)	Microemulsion	✓ excellent in vitro compatibility in physiological environment, and efficacy in cancer cells killing when exposed to MW;	MR imaging-guided MW thermotherapy.	[29]
Gelatin Ion Gels	(Emim)(EtSO_4_)	Dissolution/Photoreduction	✓ induction of antimicrobial activity by *in situ* preparation and AgNO_3_ Nps inclusion by photoreduction; ✓ IGs have self-healing properties, multiadhesive nature, reversible stretching efficiency, and high conductivity.	Not defined	[31]
	(Emim)(Cl)	Gelation	✓ more IL leads to a lower gel modulus due to the tendency of hydrophobic linkages; however, these IGs are able to recover their network structures to a higher degree during the healing process.	biomedical engineering	[92]
	(Emim)(EtSO_4_)	Dissolution/Gelation	✓ [Emim][EtSO_4_] was found to be the entrapment of GOD and HRP in gelatin type A with subsequent maturation; ✓ GOD retain up to 70% of the initial activity after storing at 4 °C for 2 weeks, while HRP retained 91% of its initial activity.	colorimetric glucose detection	[95]
Gelatin films	(Emim)(OAc)	Doping	✓ PE stable until 220 °C; ✓ PE are good ionic conductors.	smart windows and other ECD-based devices	[129]
Gelatin hydrogels	Omim·PF6	Ultrasonication	✓ immobilized HRP have higher thermal stability; ✓ better enzyme electrode performance using more hydrophobic ILs; ✓ sensitive response in the presence of H_2_O_2_.	immobilization of enzymes and fabrication of biosensors	[96]

Abbreviations: ((Bmim))((BF4))—1-Butyl-3-methylimidazolium tetrafluoroborate, ECD—electrochromic devices, (Emim)(Cl)—1-ethyl-3-methylimidazolium chloride, (Emim)(OAc)—1-ethyl-3-methylimidazolium acetate, (Emim)(Br)—1-ethyl-3-methylimidazolium bromide, (Emim) (EtSO_4_)—1-ethyl-3-methyl-imidazolium ethyl sulfate, (Omim)(Cl)—1-octyl-3-methylimidazolium chloride, OMIM·PF_6_—1-Octyl-3- methylimidazolium hexafluorophsohate, (Gly·H)Cl—glycine hydrochloride, GOD—glucose oxidase, HRP—horseradish peroxidase, IGs—ionogels, IL—ionic liquid, MW—microwave, MR—magnetic resonance, NPs—nanoparticles, PBS—phosphate-buffered saline, PE—polymer electrolyte, AgNO_3_—silver nitrate, and H_2_O_2_—hydrogen peroxide.

**Table 2:** Marine-derived blended polymers processed in different ionic liquids.

Polymer Blends	Ionic Liquid/other Reagents	Process	Improved Properties	Potential Applications	References
Chitin/ Antheraea pernyi silk fibroin based sponges	(Bmim))(OAc))	Co-dissolution/Freeze-drying	✓ sponges presented good porosity and interconnectivity values, and a considerably high swelling degree in PBS	Cartilage regeneration	[13]
Chitin-sodium alginate film	BPPF6	Solutions mixing	✓ good detection precision of H_2_O_2_ detection; ✓ sensor with improved bioactivity, storage stability, and reproducibility	biosensor	[26]
Chitin-calcium alginate fibers	(Emim)(OAc)	Microwave IL-assisted extraction/dissolution/electrospinning	✓ great in vivo outcomes, with re-epithelialization and complete coverage of the dermal fibrosis with hyperplastic epidermis after only 7 days of treatment	wound care dressings	[25]
Chitin–Cellulose Nanofibers	(Emim)(OAc)	Electrospinning	✓ Incorporation of MCC allows the preparation of materials with improved strength.	Not defined	[24]
Chitin and hydroxyapatite	(Bmim)(OAc)	Dissolution	✓ 3D porous microstructure positively influence osteoblast-like cells viability and proliferation (65%-85% porosity and of 100–300 μm pore sizes).	bone tissue engineering	[19,118]
Chitin–poly(lactic acid) Fibers	(Emim)(OAc)	Co-dissolution/wet-jet spun	✓ tensile strength and plasticity of the fibers depended on the chitin to PLA ratio;	Not defined	[130]
Chitin/SAIB scaffolds	(Bmim)(OAc)	Co-dissolution/freeze-drying	✓ different values of porosity (ranging from 52 to 85%); ✓ no cytotoxicity when culturing in vitro human adipose-derived stem cells onto the surface of the scaffolds for 72 h	tissue engineering scaffolding	[131]
Agarose/chitosan ionogels	(Bmim)(Cl)	Blending/Gelation	✓ good stability and enhanced material properties compared with individual biopolymers	quasisolid dye sensitized solar cells, actuators, sensors or electrochromic displays	[30]
Carrageenan/cellulose gels	(Bmim)(Cl)	Co-dissolution	✓ three types of carrageenans (k-, ι- and λ) were blended with cellulose; ✓ λ-carrageenan gave a better miscible composite gel		[98]
Chitosan/cellulose	(Gly·H)(Cl) and (Bmim)(Cl)	Dissolution/dry-wet spinning	✓ good mechanical strength and excellent thermal stability		[104]
	(Emim)(OAc)	Dissolution/electrospinning	✓ produce fiber films with the potential to be applied as an antibacterial and antimicrobial agent to treat skin ulcers	Wound treatment	[105]
	(Bmim)(Cl)	Co-dissolution/cast into substrate	✓ Produce composite films with the combined advantages of their components: superior mechanical strength (from CEL) and excellent adsorption capacity from CHT. ✓ They can be reused with similar adsorption efficiency.	Adsorption of microcystin LR, produced by cyanobacteria present in drinking waterWound dressings	[126,132]
Chitosan/cellulose/hydroxyapatite	(Bmim)(Cl)	Dissolution	✓ synergy of the individual properties of the used components (mechanical strength from cellulose, antimicrobial activity, and an ability to deliver active agents from chitosan)	bone tissue engineering	[27]
Chitosan/cellulose/keratin	(Bmim)(Cl)	Co-dissolution/cast into substrate	✓ Improved mechanical and thermal physical properties.	treatment of chronic and ulcerous wounds	[133]
Chitosan/chondroitin sulfate hydrogels	(Hmim)(HSO_4_)	Blending/gelation	✓ excellent stabilities (in the 1.2–10 pH range); ✓ larger swelling capacities; ✓ excellent biocompatibility upon both VERO and HT29 cells	treatment of water and wastewater	[28]
Chitosan/silk fibroin hydrogels	(Bmim)(OAc)	Blending/Gelation	✓ hydrogels have microporous, lamellar structure and viscoelastic behavior; ✓ supported the adhesion and growth of primary human dermal fibroblasts	skin tissue engineering approaches	[14]
Collagen-alginate-hydroxyapatite beads	(TEA)(OAc)	CaCl_2_-based crosslinking	✓ higher water uptake ability due to collagen addition that decreases after 5 days; ✓ successful drug loading and good antimicrobial properties; ✓ hemolysis rates below the permissible limit (5%) thereby showed hemocompatibility	bone regeneration	[122]
Collagen/ Hydroxyapatite/ Alginate	(TEA)(OAc)	Dissolution	✓ hemocompatibility, promising antibacterial properties and drug load efficacy; ✓ used as potential bone filler	treatment of deep intraosseous defects	[134]
Collagen/PVA hydrogels	(Bmim)(OAc)	Blending	✓ tensile strength in the range of 2.4 to 8.5 MPa; ✓ hemocompatible (less than 5%) without toxic effects to the blood	osteochondral patches	[135]
Gelatin/Poly(Vinyl Alcohol) films	Lidocaine–Diclofenac IL	Freeze-thawing	✓ successful physical transformation of the lidocaine–diclofenac ionic liquid drug; ✓ controlled drug release patch	transdermal patches	[97]

Abbreviations: (Bmim)(OAc)—1-Butyl-3-methylimidazolium acetate, (Bmim)(Cl)—1-Butyl-3-methylimidazolium chloride, BPPF_6—_N-butylpyridinium hexafluorophosphate, (Emim)(OAc)—1-ethyl-3-methylimidazolium acetate, (Gly·HCl)—glycine hydrochloride, (Hmim)(HSO_4_)—1-hydrogen-3-methylimidazolium hydrogen sulfate, HT29—epithelial colorectal adenocarcinoma cells, IL—ionic liquid, MCC—microcrystalline cellulose, PBS—phosphate-buffered saline, PLA—poly(lactic acid), PVA—poly(vinyl alcohol), (TEA)(OAc)—triethanolamine acetate, VERO—healthy kidney epithelial cells originated from African green monkey, 3D—three-dimensional, H_2_O_2_—hydrogen peroxide, κ—kappa, λ—lambda, and ι—iota.

## 5. Biomedical Applications of Marine-derived Polymers in Ionic Liquids

### 5.1. Wound Repair 

**Figure 7:**
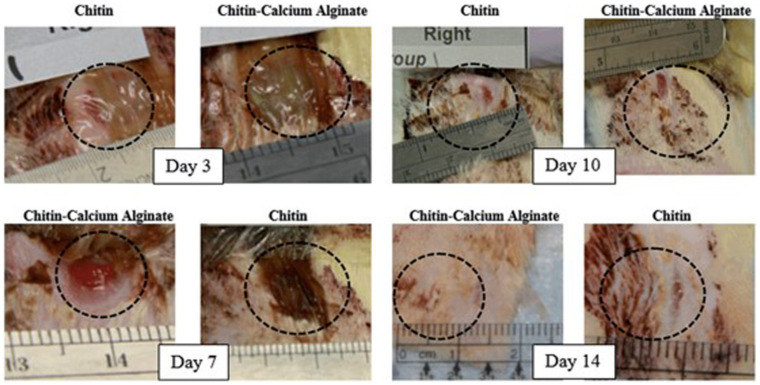
Representative images of the wound sites taken on days 3, 7, 10, and 14. Reprinted from [25]. Copyright 2017 with permission from Elsevier.

The beneficial features of marine polymers for wound healing have stimulated many studies involving the use of marine polymers/IL solutions in the production of biomaterials to be applied as support to enhance the wound-healing process [14,133]. In those approaches, the biomacromolecules combination such as chitosan/silk fibroin in (Bmim)(OAc) [14] or chitosan/cellulose/keratin in (Bmim)(Cl) [133] played a positive influence on the development of structures that showed suitable adhesion and the proliferation of human dermal fibroblasts (hDFb) and superior mechanical strength, bactericide action, and the controlled release of drugs, respectively. Roger RD et al. proposed the co-dissolution of chitin and alginate in (Bmim)(OAc), followed by extrusion of the solution into a coagulation bath to form chitin-based fibers as wound dressings [25]. Those fibers were applied on a full-dermal-thickness wound model (rat model, histological evaluation) and maintained on the wounds for up to 14 days. The wound-healing studies indicated that the chitin-calcium alginate-covered wound sites underwent normal wound healing with re-epithelization and that the coverage of the dermal fibrosis with the hyperplastic epidermis was consistently complete after seven days of treatment (Figure 7). 

The (CEL/CHT) composite films prepared using a green and totally recyclable method were also developed for wound-dressing applications, helping to promote wound healing by creating a moist microenvironment for proper tissue regeneration [132]. According to the authors, (Bmim)(Cl) was used as a single solvent to produce composites that are antibacterial, hemostatic, biocompatible, nontoxic to fibroblasts, and a good absorbent for anticoagulated whole blood and are able to maintain moisture balance for wound healing. The composites absorbed blood at the same rate and volume as commercially available wound dressings [132].

### 5.2. Bone Regeneration 

Marine-derived polymers and proteins processed through IL have been used as excellent candidates for bone/cartilage tissue engineering applications, particularly when in composites containing hydroxyapatite (HA) [27,118,122,136,137]. These composites are mechanically superior when compared to the individual components—for example, the ductility of collagen or gelatin compensates for the poor fracture toughness of hydroxyapatite, and their biological functionality is improved, presenting antimicrobial activity due to chitosan and osteoconductivity derived from HA. The addition of a ceramic compound (HA) promoted higher stability and better resistance to three-dimensional swelling and deformation. 

Many clinical applications may benefit from the incorporation of collagen to hydroxyapatite due to shape command, spatial adaptation, enhanced wall adhesion, and the potential to promote clot formation and subsequent stabilization [134,138]. Bioactive beads composed of collagen, hydroxyapatite, and alginate were prepared using a triethanolamine acetate ionic liquid as a solvent and evaluated to be used as potential bone fillers. The prepared beads showed hemocompatibility, promising antibacterial properties and drug load efficacy [122].

Chitosan/cellulose/hydroxyapatite multifunctional composites using (Bmim)(Cl) as a solvent were proposed by Mututuvari et al. [27]. The proposed composite material presented the adequate features for bone tissue engineering derived from each of the individual components, mechanical strength from cellulose, antimicrobial activity, and an ability to deliver active agents (drugs or growth factors) from chitosan.

**Figure 8:**
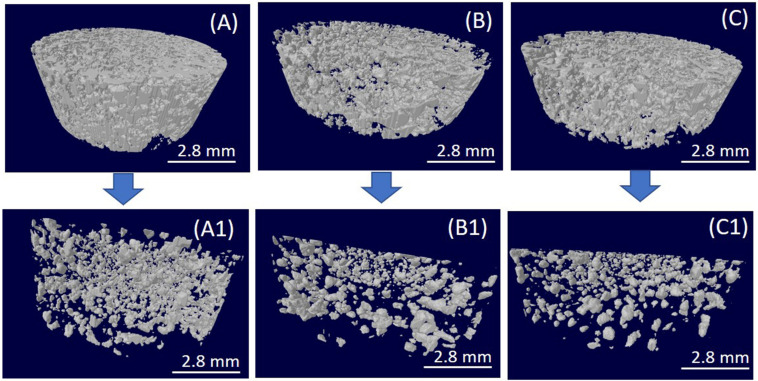
μ-microcomputed tomography of the chitin-based composite porous structure prepared using hydroxyapatite (HA): (**A**) Ch1HA, (**B**) Ch2HA, and (**C**) Ch3HA. (**A**–**C**) are complete structures, while (**A1**–**C1**) are HA-only. Modified from [118].

Chitin and hydroxyapatite composites were prepared by Silva et al. [118] using (Bmim)(OAc), achieving an enhanced dispersion of the hydroxyapatite (HA). μ-CT analysis of the chitin/HA composite showed a homogeneous distribution of the HA across the composite structure (Figure 8), where the HA content decreased with the increasing polymer concentration. The designed system has the potential to be applied for bone tissue engineering purposes, as it presented a porous microstructure (65%–85% porosity and pore sizes of 100–300 μm) able to influence osteoblast-like cells viability and proliferation [105] positively.

### 5.3. Drug and Gene Delivery 

ILs have been extensively explored in the pharmaceutical field, mainly as stabilizer agents for biomolecules, as solvents, or as part of drug carrier systems for poorly soluble drugs, such as active pharmaceutical ingredients (APIs) in IL systems (APIs-IL) [42]. Chitosan has been studied for the development of stimuli-responsive chitosan-based biomaterials in combination with several ILs [100,139]. In 2011, Hua and coworkers developed an innovative method that promoted the stimuli-responsive intravenous administration of hydrophobic drugs by combining them with chitosan via a Schiff reaction, using IL 1-butyl-3-methylimidazolium chloride ((Bmim)(Cl)) [139]. 

Following a different approach, biocompatible ILs (bio-ILs) were used to develop multiresponsive chitosan biomaterials [100]. Ammonium-based bio-ILs—namely, choline chloride and choline dihydrogen phosphate—were used to dope chitosan-based biomaterials, and the release of the ionic drug, sodium phosphate dexamethasone, was studied. The results suggested that, depending on the different ionic interactions that can be established between chitosan, chitosan/IL, and dexamethasone, it was suggested that they can be used as an electrically modulated drug release systems for iontophoretic applications [139]. The modifications of chitosan using ILs through several strategies, including grafting with polyethylenimine (PEI) in (Bmim)(Ac) or the synthesis of O-alkylated chitosan derivatives in (Bmim)(Cl), were also attempted and proved to improve its gene transfection performance [140,141]. Chitosan-based vectors proved to be noncytotoxic and have the ability of transcellular transport, since the presence of positive charges from amine groups in chitosan enables it to transport plasmid DNA (pDNA) into cells via endocytosis and membrane destability [140]. The properties of ILs as solvent should promote the selective alkylation of hydroxyl groups of chitosan without protecting its amino groups, associated with an improvement of the solubility of the derivatives in the organic solvent. Moreover, a lidocaine–diclofenac ionic liquid drug was loaded into a gelatin/poly(vinyl alcohol) transdermal patch using a freeze/thaw method [97]. The developed patch allowed to control the high drug release values of both lidocaine and diclofenac, the gelatin/poly(vinyl alcohol) patch, which, in addition, showed good stability over the study period of three months when kept at 4 °C or under ambient temperatures. The presented methodology revealed promising outcomes for improving the physicochemical and biopharmaceutical characteristics of poorly water-soluble drugs.

## 6. Conclusions and Future Trends

Over the years, ILs have been used as an important tool with high significance from technological and academic perspectives. When used in combination with marine-derived polymers, ILs provide sustainable approaches not only to promote their isolation but, also, to produce derivatives with different shapes and applications. ILs have opened up a large window of possibilities for the processing of high—added—-value biomaterials based on marine sources. Despite the clear advantages herein discussed, research on the use of ILs for the processing of marine polymers is still at an early stage. There are some persistent challenges to overcome—in particular, in the biomedical field, where the scale-up possibilities and in vitro/in vivo biocompatibility performances of the resulting matrices require additional research and investment. 

Despite the considerable volume of research on ILs, its family has been growing along the years with the development of the biocompatibility of ionic liquids (bio-ILs) as an eco- and biofriendly alternative IL family. Exciting outcomes are expected as a result of the exploitation of bio-IL contributions in this field, since they retain the features of commonly used ILs while improving their biological activity with reduced toxicity. In consequence, new strategies will emerge, and a significant boost in the use of ILs is envisioned in a broader range of fields. 

## Abbreviations

AgNO_3_	silver nitrate
(Amim)(Br)	1-allyl-3-methylimidazolium bromide
Amim	1-allyl-3-methylimidazolium
Ala	alanine
APIs	active pharmaceutical ingredients
APIs-IL	active pharmaceutical ingredients in ionic liquids systems
Arg	arginine
Bio-ILs	biocompatible IL
BPPF_6_	N-butylpyridinium hexafluorophosphate
(Bmim)	1-butyl-3-methylimidazolium
(Bmim)(OAc)	1-butyl-3-methylimidazolium acetate
(Bmmim)(OAc)	1-butyl-2,3-dimethylimidazolium acetate
(Bmim)(BF4)	1-butyl-3-methylimidazolium tetrafluoroborate
(Bmim)](C_2_OSO_3_)	1-ethyl-3-methylimidazolium ethylsulfate
(Bmim)(Cl)	1-butyl-3-methylimidazolium chloride
(Ch)(DHP)	Choline dihydrogen phosphate
(Dmim)(Cl)	1-decyl-3-methylimidazolium chloride
(Emim)(Cl)	1-ethyl-3-methylimidazolium chloride
(Emim)(OAc)	1-ethyl-3-methylimidazolium acetate
(Emim)(Br)	1-ethyl-3-methylimidazolium bromide
(Dmim)	1,3-dimethylimidazolium
(Emim) (EtSO_4_)	1-ethyl-3-methyl-imidazolium ethyl sulfate
Gly	glycine
(Gly·H)Cl	glycine hydrochloride
Glu	glutamate
GOD	glucose oxidase
HA	hydroxyapatite
H_2_O_2_	Hydrogen peroxide
HT29	epithelial colorectal adenocarcinoma cells
(Hmim)/ OAc)	1-hydrogen-3-methylimidazolium acetate
(Hmim)(HSO_4_)	1-hydrogen-3-methylimidazolium hydrogen sulfate
HRP	horseradish peroxidase
Hyp	hydroxyproline
IGs	ion gels
IL	ionic liquid;
(Mim)(OAc)	1-methylimidazolium acetate
MCC	Microcrystalline cellulose
MW	microwave
MR	magnetic resonance
(mim)	1-hydrogen-3-methylimidazolium
NPs	nanoparticles
(Omim)(OAc)	1-octyl-3-methylimidazolium acetate
(Omim)(Cl)	1-octyl-3-methylimidazolium chloride
OMIM·PF_6_	1-Octyl-3- methylimidazolium hexafluorophsohate
PBS	phosphate-buffered saline
PEI	polyethylenimine
PE	polymer electrolyte
Pro	proline
PLA	poly(lactic acid)
PVA	poly(vinyl alcohol)
(TEA)(Ac)	triethanolamine acetate
VERO	healthy kidney epithelial cells originated from African green monkey
3D	three-dimensional
Κ	kappa
λ	lambda
